# Flaxseed Lignans Alleviates Doxorubicin-Induced Premature Ovarian Insufficiency in Adult Female Mice

**DOI:** 10.1007/s43032-025-01980-x

**Published:** 2025-10-22

**Authors:** Esraa A. Elshenawy, Hend A. Mohammed, Yomna I. Mahmoud

**Affiliations:** Zoology Department, Faculty of Science, Ain Shams University, P.O. Box 11566, Abbasiya, Cairo, Egypt

**Keywords:** Premature ovarian insufficiency, Flaxseed lignans, Ovarian follicles, Estrous cycle, Pregnancy rate, Mice

## Abstract

Premature ovarian insufficiency (POI) is a condition in which the ovarian function decreases beyond the extent of the normal range of young adults’ age. This can be a consequence of cancer treatments like doxorubicin, leading to reproductive deterioration that hormonal replacement therapy cannot fully address. Flaxseed lignans are weak phytoestrogens that can mimic the effects of estrogen and alleviate POI-associated symptoms. Therefore, this study aimed to evaluate the potential of flaxseed lignans to improve the recovery from doxorubicin-induced POI in adult mice. Thirty-six regularly-cycling virgin female mice were randomly allocated into: normal, POI, and lignans-treated POI groups. These mice were assigned to either experiment I to assess cyclicity, ovarian hormones, ovarian histomorphometry, and immunohistochemistry; or to experiment II, where the females were mated to assess pregnancy rates, and embryos quantity and quality. Exposure to doxorubicin resulted in irregular estrous cycles, and significant decrease in body and ovarian weights, estradiol, follicular count, and pregnancy rate; while showed a significant increase in FSH and follicular atresia. Lignans treatment improved body and ovarian weights, and significantly ameliorated the decrease in estradiol levels, viable follicular count, as well as the pregnancy rate. Lignans have been also observed to reduce elevated FSH levels and the number of atretic follicles, while also helping to normalize estrous cycle regularity. In conclusion, flaxseed lignans, due to their estrogen-like effects, can help mitigate reproductive deteriorations caused by cancer treatments, which suggests their potential use in treating premature ovarian insufficiency.

## Introduction

The mammalian ovary encompasses follicles at different developmental stages. These follicles are the basic functional unit of the ovary, as they sustain the maturation of the oocytes and secrete the hormones that maintain the menstrual cycle and support pregnancy [1]. The number of primordial follicles in each ovary is determined during embryonic development and gradually declines through atresia before and after birth until puberty, forming what is known as the ovarian reserve [2]. Oocytes are recruited from this finite pool of primordial follicles that are usually exhausted from the ovary during mid-adult life. The compromise of this reserve during the reproductive age results in premature ovarian insufficiency (POI), which usually manifests as amenorrhea and infertility, and is diagnosed by high levels of follicle-stimulating hormone (FSH) and low levels of estradiol (E2) and inhibin [3]. POI is a well-documented long-term side effect of cancer chemotherapy, and it is a significant contributor to new cases of POI among women of reproductive age, accounting for 20–80% of cases. Of these chemotherapeutic drugs is doxorubicin (DOX), which is commonly-used to treat cancers of the breast, ovary, bladder, and uterus [4]. It primarily works by generating reactive oxygen species and disrupting mitochondrial function, which impairs DNA replication, and ultimately kills cancer cells [5]. Specifically, DOX exerts its cytotoxic effects by stabilizing the DNA-topoisomerase II cleavage complex, preventing religation and causing persistent DNA breaks. This activates DNA damage and induces apoptosis, particularly in rapidly dividing cancer cells [6]. However, DOX also affects normal, rapidly proliferating cells such as those of the ovary, causing significant side effects [7]. Because of this off-target effect on highly-proliferating ovarian cells, we used DOX in this study as an agent to induce cytotoxicity to the highly-proliferating ovarian cells, the granulosa cells. The damage of these estrogen-producing cells leads to follicular atresia and consequently, POI.

Hormone replacement therapy (HRT) has been used to treat POI. However, such treatment is palliative, and although it has the potential to enhance the quality of life for women, it lacks the ability to reinstate ovarian functions like follicular growth, hormone secretion, or ovulation [8]. Moreover, the long-term use of HRT is related to an increased risk of endometrial hyperplasia and ovarian cancer, as well as breast cancer and cardiovascular events [9, 10]. For this reason, researchers began to investigate alternatives to HRT, such as phytoestrogens, which are relatively safe plant secondary metabolites, cost-effective, and easily administered in a non-invasive manner [11, 12].

Phytoestrogens are plant-based estrogen-like compounds [13]. Strong phytoestrogens, such as isoflavones found in soy products, may cause thyroid disruption and stimulation of estrogen-dependent breast cancer cell growth, especially with prolonged exposure [14]. Coumestans, show strong estrogenic activity that may negatively affect reproductive health, and subsequently resulting in ovulation failure [15]. Stilbenes exhibit weaker estrogenic effects but may interact with medications such as anticoagulants at high doses [16]. Unlike other classes of phytoestrogens, lignans are generally well-tolerated and not associated with the adverse effects of other classes of phytoestrogens, which highlights their potential as a safer class of phytoestrogens and a promising option for protecting against conditions like POI. Further supporting their safety and physiological activity, [17]. reported that mice with cyclophosphamide/busulfan -induced POI, when treated orally with the lignan derivative secoisolariciresinol diglucoside (SDG) at doses of 50, 100, or 200 mg/kg daily for four weeks, showed marked recovery of ovarian function and exhibited no adverse clinical signs, demonstrating both the efficacy and tolerability of the compound under the experimental conditions. Additionally, [18]. reported that normal mice treated orally with flaxseed lignans at a dose of 200 mg/kg daily for four weeks exhibited no adverse clinical signs confirming that flaxseed lignans are well tolerated under physiological conditions.

Moreover, lignans can directly neutralize free radicals by donating electrons to stabilize them, thereby preventing oxidative damage to cells and biomolecules [19]. Certain lignans can modulate gene expression related to antioxidant defense mechanisms, leading to increased synthesis of antioxidant enzymes and proteins [20]. In addition, lignans exerts anti-apoptotic, and anti-inflammatory which may be an interesting alternative therapy for POI [21]. Although clinical data on lignans in POI is limited, their antioxidant and cardioprotective effects have been documented in broader populations, suggesting potential benefits in reducing cardiovascular and bone-related risks. Unlike synthetic estrogen, lignans do not require progestogen co-administration and are generally well tolerated, even at high intake levels. Moreover, unlike hormone therapy, lignans do not increase clotting risk in postmenopausal women, thus supplements may serve as a treatment option for patients who have contraindications to hormone therapy [22].

However, the protective role of flaxseed lignans is tissue-selective as reported by [23]. who demonstrated that flaxseed mitigates chemotherapy-induced toxicity in non-cancerous tissues, while it preserves the cytotoxic effects of DOX on tumor cells. Although no previous studies have specifically investigated the chemical interaction between lignans and DOX, but the study of [24]. explored the potential of the flaxseed-derived lignan, secoisolariciresinol (SECO), to reverse drug resistance in doxorubicin-resistant NCI/ADR-RES cancer. The findings revealed that SECO significantly enhanced the cytotoxic effect of DOX compared to DOX alone, indicating its potential to re-sensitize resistant cancer cells and improve the overall efficacy of chemotherapy. Therefore, this study aimed to evaluate the potential of flaxseed lignans to improve the recovery from chemotherapy DOX-induced POI in adult mice, which has never been studied before.

## Materials and Methods

### Chemicals and Reagents

Flaxseed lignans capsules (organic natural flaxseed hulls) were purchased from Lignans for life Company (USA). Doxorubicin HCL (Adricin^®^, 50 mg/25 ml) was purchased from Hikma Specialized Pharmaceuticals Company (Badr city, Cairo, Egypt). Commercial kits for determining the hormones FSH and E2 were from DRG international Inc. (USA). The antibodies of the nuclear protein Ki67, cleaved caspase 3, ER-α and inhibin were from Dako (Carpinteria, CA, USA). All other chemicals used in the current study were of analytical grade. All solutions and reagents were prepared immediately before use.

### Animals

Adult virgin female albino mice of CD1 strain, 8 weeks old and weighing 22–25 g, were purchased from the Schistosoma Biological Supply Program Unit, Theodore Bilharz Research Institute (Giza, Egypt). Mice were transported to the animal care facility of Zoology Department at Faculty of Science, Ain Shams University, one week prior to the initiation of the experiments for acclimatization to the laboratory conditions to eliminate the effect of stress. The animals were housed under hygienic conditions in polypropylene cages bedded with clean wood shaving. Good ventilation, a 12:12 h light/dark cycle, a temperature of 23–25 °C and 45–55% relative humidity were maintained. The animals were allowed free access to water and standard rodent pellets. All animal experiments comply with the US National Institutes of Health guide for the care and use of Laboratory animals (NIH Publications No. 85 − 23, revised 1996). In addition, this protocol was approved by the Ethical Review Board of Faculty of Science, Ain Shams University (Cairo, Egypt), with the protocol number ASU-SCI/ZOOL/2025/1/2.

### Vaginal Smear Collection

The estrous cycle of each mouse was examined daily between 8:00 — 10:00 am for 12 days (3 cycles in normal mice) before the beginning of the experiment to assess cycle regularity, using the swab smear technique [25]. Based on the vaginal smear cytology and proportion of vaginal epithelial cells, cornified epithelial cells and leukocytes, the estrous cycle of rodents was divided into four stages as described by [25]. Prolonged cycles were defined as those remaining in the same phase for 4 days or more. Cycles, in which the alternation among the phases did not follow the sequence proestrus, estrus, metestrus and diestrus, were considered irregular. After selecting regularly-cycling animals for the experiment, daily vaginal smears were also traced during the whole experiment to assess cycle patterns (regularity and length) in POI experimental mice.

### Experimental Design

A total number of 36 regularly-cycling female mice were chosen for the study and were randomly allocated into 3 groups (*n* = 12) as follows: Normal group: negative control mice that were neither treated with lignans nor injected with doxorubicin; POI group: mice that were injected intraperitoneally with a single dose of doxorubicin (20 mg/kg in 0.9% saline) [26], and were observed for 21 consecutive days; and lignans-treated POI group: mice that were orally-treated with flaxseed lignans (200 mg/kg in distilled water) [18] using a stomach tube a week before doxorubicin injection, then continued for 21 consecutive days after doxorubicin injection. This dose is equivalent to the human recommended dose (1 g/day) [27].

Mice in the negative control and POI group received the vehicle of lignans-treated group at the same volume and route; while the negative control group only was injected with the vehicle of the POI-induced groups. Animals were inspected daily for any clinical symptoms, and their body weight was recorded at the beginning and the end of the experiment, and their weight change was calculated.

The animals were assigned to either Experiment I (*n* = 7/group) to assess cyclicity, POI-related hormones, ovarian histomorphometry and immunohistochemistry; or to Experiment II (*n* = 5/group), where the females were mated to assess the pregnancy rates, embryos quantity and quality.

### Experiment I

#### Gross Morphology

The mice were weighed individually at the beginning of the experiment and at its end, just before collecting blood and tissue samples to determine the body weight change.

#### Sample Collection

At the end of the experiment and after 21 days of doxorubicin injection, seven mice from each group were selected if in the metestrus phase, then fasted overnight, weighed, and anesthetized with diethyl ether. Blood samples were collected from their retro-orbital venous plexus using capillary tubes. Two samples per mouse were collected in clean centrifuge tubes without EDTA, allowed to stand at room temperature for 45 min, and then centrifuged at 6000 rpm for 15 min at 4 °C to separate the serum. The serum was stored at − 20 °C in sterile containers for hormonal assays. Finally, the mice were necropsied, and their ovaries were excised, weighed and processed for histological and immunohistochemical investigations.

#### Hormonal Assays

Serum levels of FSH and E2, were estimated using enzyme-linked immunosorbent assay (ELISA) kits (DRG international Inc., USA), according to the manufacturer’s instruction.

#### Histomorphometric Studies

The ovaries were cut into halves before immersing them in 10% neutral buffered formalin for 48 h. After that, the ovaries were washed overnight in running tap water, followed by dehydration in an ascending series of alcohol, clearance in terpineol, and eventual embedding in paraffin wax. Mid sections were cut at 5-µm thickness, dewaxed, hydrated, and stained with hematoxylin and eosin for routine histology, or using Masson’s trichrome to evaluate the ovarian cortex architecture and differentiate collagen on tissue sections. Stained sections were dehydrated through ascending series of ethyl alcohol, cleared in xylene and finally mounted with DPX. Sections were photographed by a camera attached to a Leica DM LS2 microscope (Leica Microsystems, Germany) at Faculty of Science, Ain Shams University. The data were calibrated automatically to convert the measurement units (pixels) produced by the image analyzer program into actual micrometer units. The middle section of each ovary in each group was examined to quantitatively assess the count of different types of viable ovarian follicles, as well as the count of atretic follicles and corpora lutea.

#### Immunohistochemical Procedure

Immunohistochemical detection for the investigated markers: nuclear protein Ki67, cleaved caspase 3, ER-α and inhibin, was performed on ovarian Sect. (5-µm thick) using avidin–biotin–peroxidase technique according to the manufacturer’s protocol. Positive immunoreactions appeared as brown coloration. To make certain reproducibility, 5 animals from every group were examined and their sections were processed concurrently on the same slide.

#### Statistical Analyses

Numerical data are reported as mean values and standard error of mean. GraphPad Prism (GraphPad software, San Diego, CA, USA) was used to conduct all statistical analysis. Data were analyzed statistically using One-way ANOVA followed by post hoc multiple comparisons (Tukey’s test) for comparative analysis between the groups. *P* < 0.05 was regarded as statistically significant.

### Experiment II

#### Mating and Pregnancy Rate

The pregnancy rate was calculated as the number of pregnant mice to the number of females mated. Pregnancy was obtained by housing a fertile-proven male mouse with two virgin females at the estrous phase overnight. Insemination was confirmed by the presence of vaginal plug or spermatozoa in the vaginal smear. The day of mating was considered zero day of pregnancy. According to the Mouse Breeding Colony Management Guidelines, female mice that were continuously housed with a proven fertile male for four weeks and did not become pregnant were sacrificed, and their pregnancy rate was recorded as 0 [28].

#### Examination of Mothers and Fetuses

The maternal body weight of mice were recorded at the 1 st, 7th, 14th and 18th days of gestation. The assured pregnant mice were sacrificed on day 18 of pregnancy. During necropsy, the uterine horns were weighed and then opened to examine the number of implantation sites, and live or dead/resorbed fetuses. The fetuses were examined carefully for any morphological malformations using a binocular stereo-microscope. The body length and weight of the fetuses, in addition to t-he placental weight were recorded. Also, the placental weight was recorded.

## Results

### Experiment I

#### Estrous Cycle

**Fig. 1:**
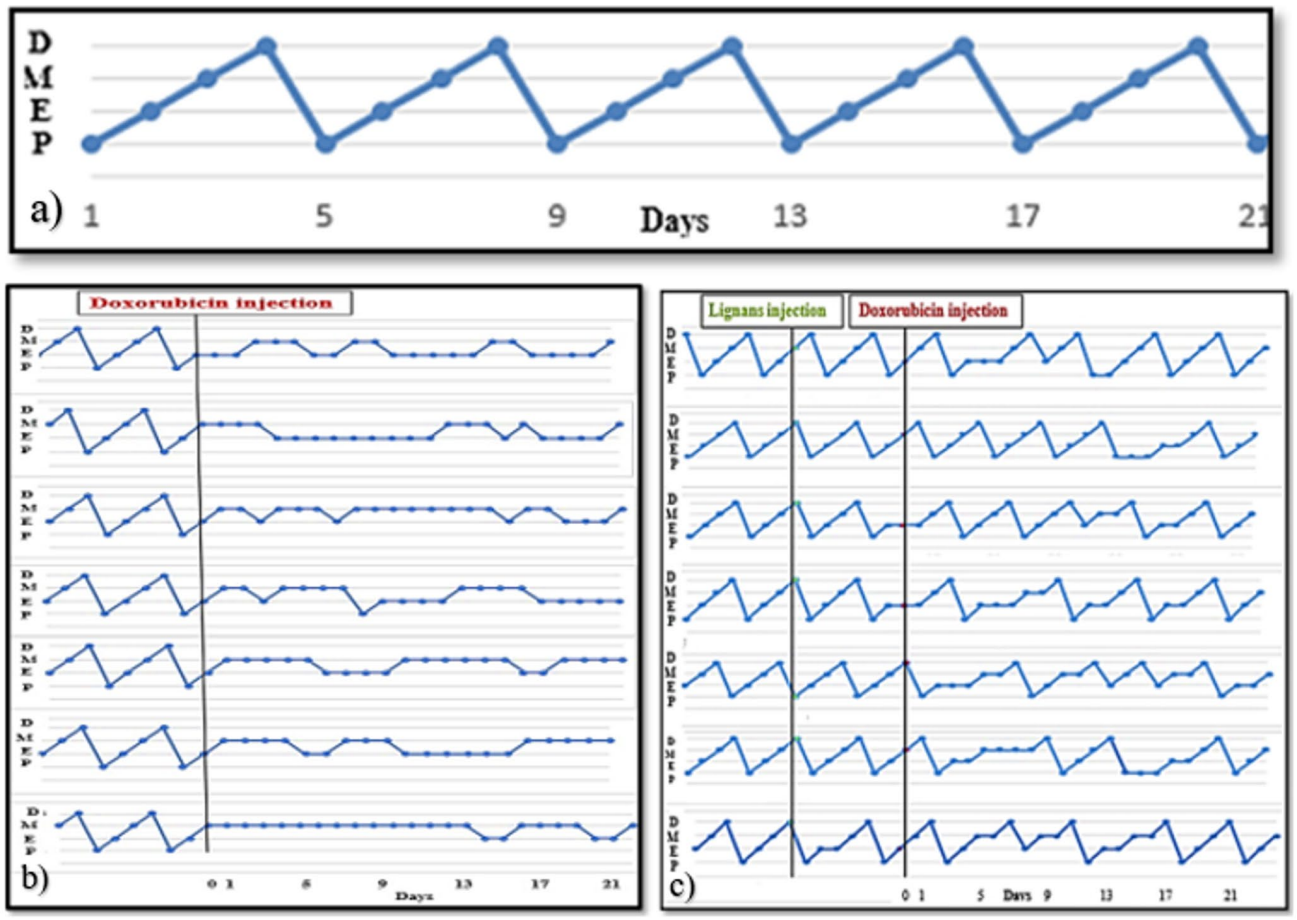
Effect of flaxseed lignans on POI mice. (**a**) Normal; (**b**) POI; and (**c**) lignans-treated POI group. Y-axis represents estrous cycle stages, *D* diestrus; *M* metestrus; *E* estrus; *P* proestrus

Based on the vaginal smears, normal mice exhibited regular estrous cycles (Fig. 1a) with normal duration of each stage during the whole experiment (Table 1). On the other hand, POI mice showed irregular estrous cycles (Fig. 1b), with a significant increase in the incidence of metestrus and estrus stages and complete absence in the proestrus and diestrus stages (Table 1). However, mice with lignans almost restored cycle regularity (Fig. 1c; Table 1).

**Table 1:** Effect of flaxseed lignans on the percentages of estrous cycle stages in POI adult female mice

		Groups		
		Normal	Premature ovarian insufficiency	Premature ovarian insufficiency + lignans
Estrous cycle phases %	Proestrus	25.0 ± 0.00	0.64 ± 0.64**	24.7 ± 0.68 ##
	Estrus	25.0 ± 0.00	43.7 ± 6.66*	29.1 ± 2.02
	Metestrus	25.0 ± 0.00	55.5 ± 6.78**	24.7 ± 1.23##
	Diestrus	25.0 ± 0.00	0.00 ± 0.00**	21.4 ± 1.22*##

Values are expressed as means + SEM. Data were analyzed statistically using One-way ANOVA followed by post hoc multiple comparisons (Tukey’s test) for comparative analysis between the groups * indicates the significant difference of all groups vs. normal group, *P<0.05, **P<0.001 # indicates the significant difference of lignans treated group vs. POI control group, ##P<0.001

#### Body Weight Change, Ovarian Gross Morphology and Relative Weight

**Fig. 2:**
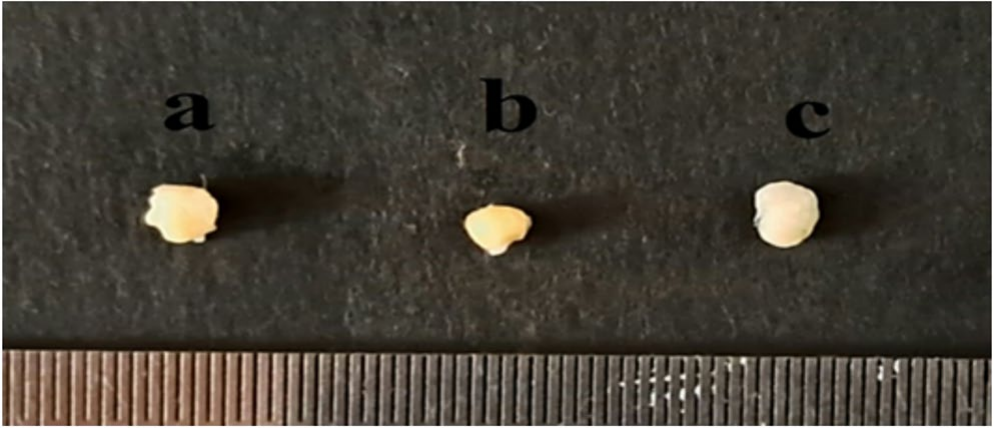
Effect of flaxseed lignans on ovarian gross morphology in POI mice (**a**) Normal, (**b**) POI control, and (**c**) Lignans-treated POI group

At the end of the experiment, normal mice significantly gained weight, in contrast to doxorubicin-injected mice that displayed significant weight loss. Lignans-treated mice showed significant weight gain compared with doxorubicin-injected mice; however, such weight gain was not comparable to that of the normal group. The ovaries of POI control group were smaller in size and less in their ovarian relative weight compared with those of the normal group. On the contrary, the ovarian size and relative weight of lignans-treated mice were comparable to those of normal mice (Fig. 2; Table 2).

**Table 2:** Effect of flaxseed lignans on the body weight change, ovarian relative weight and serum levels of follicle-stimulating hormone and estradiol of POI mice

	Groups		
	Normal	Premature ovarian insufficiency	Premature ovarian insufficiency + lignans
Body weight change %	9.56 ± 0.96	−12.44 ± 2.19**	2.33 ± 2.07*^##^
Ovarian relative weight%	0.03 ± 0.003	0.02 ± 0.001*	0.03 ± 0.007
Follicle-stimulating hormone (mIU/mL)	0.07 ± 0.01	0.74 ± 0.09**	0.07 ± 0.01##
Estradiol (pg/mL)	8.80 ± 0.58	0.57 ± 0.10**	3.92 ± 1.72##

Values are expressed as means + SEM. Data were analyzed statistically using One-way ANOVA followed by post hoc multiple comparisons (Tukey’s test) for comparative analysis between the groups, * indicates the significant difference of all groups vs. normal group, *P<0.05, **P<0.001.# indicates the significant difference of lignans-treated group vs. POI control group, ##P<0.001

#### Hormonal Results

In doxorubicin-injected mice, serum levels of FSH were significantly high, whereas serum levels of E2 were significantly low in comparison with those of the normal group. However, lignans treatment resulted in significant decrease in the elevated levels of FSH, and significant increase in the decreased levels of E2 in comparison with those of doxorubicin-injected mice (Table 2).

#### Histological Results

**Fig. 3:**
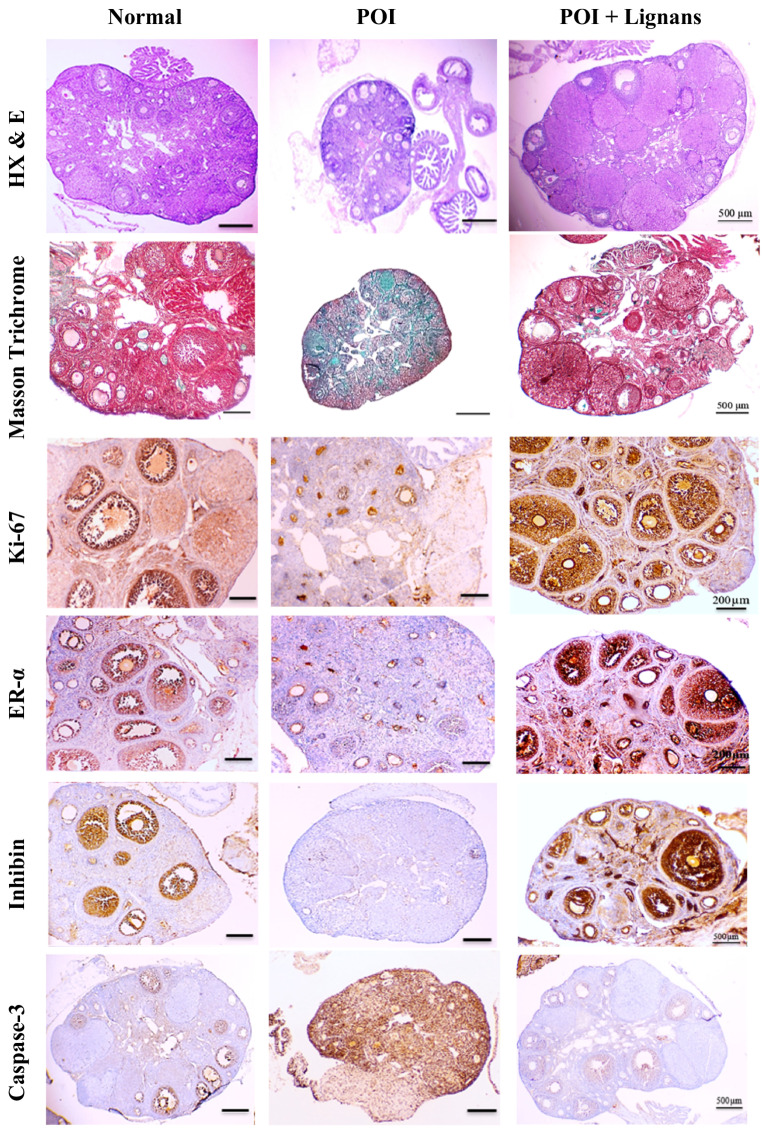
Photomicrograph of the effect of flaxseed lignans on the histology and immuno-histochemical expression of Ki-67, ER-α, inhibin and caspase-3 in ovarian tissue of POI mice

The ovarian tissue is normally divided into an outer cortex encompassing follicles at different stages of development and an internal medulla of loose connective tissue and many blood vessels. The ovarian follicles included primordial, primary, secondary, and antral follicles, corpora lutea, and few atretic follicles (Table 3; Fig. 3), with no signs of fibrosis (Fig. 3). On the other hand, morphometric analysis of the follicular population of the ovaries of POI control group revealed significant reduction in the numbers of the most of the viable follicles, and a significant increase in the number of atretic follicles compared to normal mice (Table 3; Fig. 3). These histological alterations were accompanied with increased fibrous deposition around the atretic follicles and the medullary blood vessels (Fig. 3). Treating POI mice with lignans induced an increase in the count of all types of viable ovarian follicles and corpora lutea, and a decrease in the number of atretic follicles, in addition to lack of fibrosis (Table 3; Fig. 3).

**Table 3:** Effect of flaxseed lignans on the follicular count in POI mice

		Groups		
		Normal	Premature ovarian insufficiency	Premature ovarian insufficiency + lignans
Follicular count	Primordial follicles	7.00 ± 0.48	1.00 ± 0.43**	5.28 ± 1.10#
	Primary follicles	2.57 ± 0.57	0.00 ± 0.00**	1.85 ± 0.45#
	Secondary follicles	2.57 ± 0.57	0.00 ± 0.00**	1.85 ± 0.67#
	Tertiary follicles	3.28 ± 0.35	0.00 ± 0.00**	2.57 ± 0.48##
	Corpus luteum	5.85 ± 0.59	0.57 ± 0.42**	6.28 ± 1.93#
	Total viable follicles	21.28	1.57	17.75
	Atretic follicles	6.42 ± 0.99	68.14 ± 5.34**	9.28 ± 1.37##

Values are expressed as means + SEM. Data were analyzed statistically using One-way ANOVA followed by post hoc multiple comparisons (Tukey’s test) for comparative analysis between the groups, * indicates the significant difference of all groups vs. normal group, **P<0.001 # indicates the significant difference of lignans-treated group vs. POI control group, #P<0.05, ##P<0.001

#### Immunohistochemical Results

##### Ki-67

Ki-67 is a cellular marker for proliferation, a nuclear antigen specifically expressed during the late G1, S, M and G2 phases of the cell cycle (but not G0). Therefore, its expression is used as an indicator of cell viability. Ovarian sections of normal mice showed strong and distinct Ki-67-immunoexpression in the nuclei of granulosa cells of viable follicles. Cells of newly formed corpora lutea were also Ki-67 positive, while those of older ones were Ki-67 negative. On the contrary, sections of POI mice showed negative Ki-67-immunoexpression in the nuclei of granulosa cells of atretic follicles. Sections of lignans-treated group showed strong Ki-67 immunoreaction at the nuclei of granulosa cells of viable follicles (Fig. 3).

##### Estrogen Receptor Alpha (ER-α)

Estrogen binds to ERs to regulate estrous cycles and reproduction. The expression of ER-α is an indicator of estrogen signal transduction in the ovary. Ovarian sections of normal mice showed strong ER-α -immunoexpression in the nuclei of granulosa cells of viable follicles. On the contrary, sections of POI mice showed negative ER-α -immunoexpression in the nuclei of granulosa cells of atretic follicles. Sections of lignans-treated group showed strong ER-α immunoreaction at the nuclei of granulosa cells of viable follicles (Fig. 3).

##### Inhibin

Inhibin is an ovarian hormone that regulates the secretion of pituitary FSH during the cycle through a feedback mechanism, and is considered as a marker for ovarian reserve and follicular development. In the ovaries of the normal group, the immunohistochemical expression of inhibin in granulosa cells of large follicles was moderate. On the other hand, ovaries of POI group showed negative inhibin reactivity in atretic follicles. Sections of lignans-treated group showed strong inhibin immunoreaction at the cytoplasm of granulosa cells of viable large follicles (Fig. 3).

##### Cysteine Aspartate-specific Protease (Cleaved Caspase-3)

Cleaved caspase-3 is a marker for apoptosis, which is prevalent during ovarian follicular atresia. Caspase-3 is normally localized in the cytoplasm whereas the nucleus contained little or no staining for this enzyme. When activated during apoptosis, it moves to the nuclei of apoptotic cells. The immunoreactivity of caspase-3 in the ovaries of normal mice was weak to moderate. Ovaries of doxorubicin-injected mice showed strong caspase-3 immunoreactivity. Sections of lignans-treated group showed weak immunostaining for caspase-3 (Fig. 3).

### Experiment II

#### Pregnancy Rate

Normal mice showed 100% pregnancy rate, while none of doxorubicin-injected mice group became pregnant (pregnancy rate = 0%). Flaxseed lignans-treated mice showed a significant increase in the pregnancy rates (60%) compared to those of POI mice.

#### Mother Weight

All pregnant female mice of Normal and lignans-treated groups showed steady increased in body weight, on the other hand doxorubicin-injected mice group (POI) did not show any signs (vaginal bulge or vaginal smear) of pregnancy at all for all individuals of the group, but a decrease in the weight of the mice in this group was observed (−3.2 ± 0.81) during the days of the experiment (Table 4).

**Table 4:** Effect of flaxseed lignans on the mother weight, and on the number, body length, body weight and placental weight of 18-day-old fetuses in POI mice

	Groups		
	Normal	Premature ovarian insufficiency	Premature ovarian insufficiency + Lignans
Mean body weight of mother (g) at zero day	29 ± 1.26	26.7 ± 0.30	29 ± 0.57
Mean body weight (g) of mother at 18th day of gestation	48.3 ± 3.20	23.5 ± 0.75**	47 ± 0.57##
Mean change of mother weight	19.3 ± 3.2	−3.2 ± 0.81**	18 ± 0.81##
Number of fetuses	11 ± 0.77	0 ± 0.00**	6 ± 2.47#
Fetus length average (cm)	2.26 ± 0.09	0 ± 0.00**	2.20 ± 0.17##
Fetus weight average (g)	1.36 ± 0.08	0 ± 0.00**	1.31 ± 0.00##
Placental weight (g)	0.13 ± 0.005	0 ± 0.00**	0.13 ± 0.009##

Values are expressed as means + SEM. Data were analyzed statistically using One-way ANOVA followed by post hoc multiple comparisons (Tukey’s test) for comparative analysis between the groups,* indicates the significant difference of all groups vs. normal group, *P<0.05, **P<0.001, # indicates the significant difference of lignans-treated group vs. POI control group, #P<0.05, ##P<0.001

#### Pregnancy Outcome

POI mice did not get pregnant and consequently did not give birth to fetuses. Lignans-treated group showed comparable normal number of 18-days old fetus, and non-significant decrease in the mean body length and weight of fetuses compared with that of the normal group. There was no difference in the placental weight on day 18 of pregnancy of the lignans-treated group compared with that of the normal group (Table 4).

## Discussion

POI is one of the adverse effects of cancer chemotherapy treatments such as doxorubicin [29]. In this study, DOX-injected mice displayed significant body weight loss. Such weight loss could be attributed to the ability of doxorubicin to disrupt several metabolic pathways, reduce lipogenesis, and hinder adipogenesis [30]. Similar results have been reported by Favreau-Lessard et al. [31]. after exposing experimental animals to doxorubicin.

Histologically, the ovaries of doxorubicin-injected mice suffered from increased follicular atresia, accompanied by medullary fibrosis indicating the establishment of POI. Similar histological results were reported by [32, 33]. The direct effect of chemotherapy-induced oxidative stress negatively affects the mitochondrial function and interferes with the balance of pro- and anti-apoptotic molecules in ovarian cells [34], resulting in follicular atresia within few hours after doxorubicin injection [35]. The present study also demonstrated that DOX-injected mice showed highly significant decrease in the number of corpora lutea as reported earlier [33]. As a result of increased follicular atresia and decreased number of corpora lutea, DOX-injected mice displayed highly significant ovarian weight loss as observed herein and elsewhere [36].

Since granulosa cells are the main site for the production of estrogen, apoptosis of these cells and hence follicular atresia makes the antral follicles unable to make estrogen [37]. Estrogen deficiency triggers a compensatory response by the hypothalamus-pituitary-gonadal axis, resulting in an elevation of FSH levels [38]. This explains the results of the current study where DOX-injected mice had significant decrease in E2 levels and significant increase in FSH levels in comparison with the mean values of the normal group. This is in line with the results of Niringiyumukiza et al. [32]. Accordingly, the estrous cycles of DOX-injected mice were irregular and did not follow the classic sequence of the estrous cycle phases. The diestrus II phase was also missing from the estrous cycles of POI mice, which is correlated with low number of corpora lutea and consequently the low concentration of progesterone, which cannot suppress the release of gonadotropins (FSH and/or LH) from the anterior pituitary [39]. The cycles were also characterized by the absence of the proestrus phase. This is probably attributed to the decrease of viable antral follicles and consequently E2 deficiency as confirmed histologically and biochemically. Similar results have been reported by Nishi et al. [40].

The disruption in the ovarian follicles significantly decreases the pregnancy rates. Moreover, hormonal disruptions and irregular estrous cycles also have profound negative effects on the fertility and pregnancy rates in female animals [41]. All of the previously-mentioned results explain the infertility of DOX-injected animals of the current study. Roti Roti et al. [42]. reported that doxorubicin-induced ovarian damage was correlated with significantly increased infertility index in experimental animals.

So far, there are no proven treatments that could increase the ovarian activity, ovulation rate, and the possibility of conception [43]. Therefore, this study aimed to evaluate the potential of flaxseed lignans to improve the recovery from chemotherapy DOX-induced POI in adult mice, which has never been studied before. In this study, mice with flaxseed lignans showed a significant weight gain compared with that of POI mice. Pourjafari et al. [44]. observed that the flaxseed treatment increased the body weight of experimental animals. Parikh et al. [45]. attributed such weight gain to the omega-3 fatty acids content in flaxseed that supports a healthy metabolism. Flaxseed lignans treatment also prevented follicular atresia observed the POI group. This could be attributed to the antiapoptotic and proliferative effects of lignans on the follicular cells as confirmed by the decrease of the activated caspase-3 and the increase of Ki-67 immuno-expressions. Our histomorphometric results are in line with the results of Jelodar et al. [46]., who observed that the flaxseed treatment increased the number of preantral and antral follicles, in addition to corpus luteum in a polycystic ovary syndrome-induced rat model. Similarly, Abd El-Galil and Mohammed [47] showed that flaxseeds efficiently alleviated the ovarian damage and restored healthy ovarian follicles in a letrozole-induced polycystic ovary syndrome rat model. They attributed this ameliorative effect of flaxseeds to their antioxidant and anti-apoptotic activities via its high contents of vitamin E, α-linolenic acid or omega-3 fatty acids, flavonoids and phenolics of flax lignan complex. Furthermore, He et al. [48]. mentioned that secoisolariciresinol diglucoside, which is the most important component of flaxseed lignans, can significantly increase the number of growing follicles in senile female mice. The authors accredited this effect to the improved expression of FSH receptor protein in granulosa cells, and to the improved absorption and transportation of nutrients. Moreover, Zhang et al. [17]. demonstrated that oral administration of SDG at doses of 100 and 200 mg/kg in a cyclophosphamide-induced POI mouse model significantly restored the number of primordial, primary, secondary, and antral follicles, and attributed its ameliorative effect to its potent antioxidant and anti-apoptotic activities, together with the ability to activate the PI3K/Akt pathway, thereby promoting granulosa cell survival and supporting follicular development.

As a result of the inhibition of follicular atresia and the increase in viable growing follicles and corpora lutea, the ovarian relative weight of lignan-treated POI mice significantly increased. Our results align with those of Paul et al. [49]., who demonstrated that flaxseed treatment restored the normal size of ovaries, increased the number of the ovarian follicles at different developmental stages, and regenerated the granulosa cells in a lead-induced toxicity rat model. Similarly, Chen et al. [50]. reported that the lignan Honokiol significantly increased the ovarian weight compared to that of the aging control group, and attributed this to the antioxidant activity of lignans, which prevents follicular atresia. Consistently, Zhang et al. [17]. showed that oral administration of SDG at doses of 100 and 200 mg/kg effectively alleviated the reduction in ovarian weight in a cyclophosphamide-induced POI mouse model, reinforcing the evidence for the ameliorative role of lignans against chemotherapy-induced ovarian damage.

Flaxseed lignans administration also decreased follicular atresia, thereby preserved granulosa cells. As granulosa cells increase their production of estrogen, as observed in the current hormonal results and the immune-expression of the estrogen receptors, which are known to mediate estrogen effects [51], and preserve the differentiation of ovarian granulosa cells, promoting follicle and oocyte growth and development, and supporting ovulation function [52]. Similarly, Zhang et al. [17]. demonstrated that oral administration of SDG at doses of 100 and 200 mg/kg in a cyclophosphamide-induced POI mouse model markedly reduced follicular atresia, which was inked to the activation of the PI3K/Akt signaling pathway, thereby promoting granulosa cell proliferation and enhancing follicular survival.

Estrogen is also known to exert negative feedback on the pituitary gland, leading to a reduction in the secretion of FSH. At the end of follicular phase, a surge in LH is triggered by high levels of estrogen. This LH surge is crucial to trigger ovulation [53], after which the remains of the mature follicle become the corpus luteum that secretes inhibin, which further suppresses FSH production. This complicated hormonal interplay ensures the proper maturation of follicles and the regulation of the estrous cycle [53]. Thus, and as a result of the reduction of follicular atresia, treatment with flaxseed lignans significantly increased the decreased levels of E2 and inhibin, and significantly decreased the elevated levels of FSH in comparison with those of POI mice in the current study. Tanideh et al. [54]. also reported that flaxseed oil elevated E2 in ovariectomized adult rats and proposed that flaxseed oil is a potential HRT treatment because it can mimic the effects of estrogen. Moreover, Abouelmagd and Hassan [55] mentioned that flaxseed oil supplementation to ovariectomized rats resulted higher estradiol levels and lower FSH levels when compared to those of ovariectomized group. Zhang et al. [17]. showed that SDG upregulated follicle-stimulating hormone receptor (FSHR) expression in granulosa cells, suggesting that lignans not only normalize circulating gonadotropin levels but may also enhance ovarian sensitivity to FSH.

As a result of the restoration of the hormonal balance, mice of the lignans-treated group almost restored the cycles’ regularity. Abd El-Galil and Mohammed [47] mentioned that flaxseed-treated groups showed restoration of the normal cycle in a letrozole-induced polycystic ovary syndrome rat model. This improvement may be attributed to the direct antioxidant and free radical scavenging activities, along with the anti-inflammatory, and anti-apoptotic properties of flaxseed. In addition, lignans-treated POI mice also showed a significant increase in the pregnancy rates compared to those of doxorubicin-injected mice. In this regard, Dutra et al. [56]. reported that a diet incorporating flaxseed resulted in an increase in both the quantity and the morphological quality of goat embryos. Didarkhah et al. [57]. reported that feeding ewes with diets containing 10% and 12% flaxseed led to enhanced reproductive performance.

Maternal body reduction can have significant implications for both the placental weight and ultimately, the fetal development [58]. The placenta plays a crucial role in supplying nutrients and oxygen to the developing fetus, and any alterations in its weight can disrupt this vital supply chain [59]. As maternal weight decreases, the placental weight may also diminish, impacting its ability to adequately support fetal growth and development. Consequently, this reduction in placental function can lead to a decrease in fetal weight and length [60]. In this study, lignans prevented fetus weight reduction during pregnancy. This effect is attributed to their ability to support overall maternal health, including optimizing nutrient absorption and promoting a favorable environment for fetal and placental development.

It is important to note that our findings are consistent with those of Zhang et al. [17]., who showed that when treatment was continued for four weeks after induction, POI caused by a single intraperitoneal injection of cyclophosphamide (120 mg/kg) combined with busulfan (30 mg/kg) was significantly reduced by the flaxseed-derived lignan SDG, given orally at 50, 100, or 200 mg/kg. Nevertheless, we used doxorubicin (20 mg/kg, i.p.) to induce POI and 200 mg/kg of flaxseed lignans orally for four weeks in a row, beginning one week prior to the doxorubicin injection and continuing for three weeks following induction. The outcomes of both studies consistently show that lignans can reverse chemotherapy-induced POI and restore reproductive function, despite these variations in induction methodology and treatment design.

In conclusion, flaxseed lignans, due to their estrogen-like effects, can help mitigate reproductive deteriorations caused by cancer treatments, which suggests their potential use in treating premature ovarian insufficiency.

## References

[1] Xiao S, Zhang J, Liu M, Iwahata H, Rogers HB, Woodruff TK. Doxorubicin has dose-dependent toxicity on mouse ovarian follicle development, hormone secretion, and oocyte maturation. Toxicol Sci. 2017;157:320–9. 10.1093/toxsci/kfx047. 28329872 10.1093/toxsci/kfx047 PMC6074798

[2] Park SU, Walsh L, Berkowitz KM. Mechanisms of ovarian aging. Reproduction. 2021;162(2):R19-33. 10.1530/REP-21-0022. 33999842 10.1530/REP-21-0022 PMC9354567

[3] Jiao X, Meng T, Zhai Y, Zhao L, Luo W, Liu P, et al. Ovarian reserve markers in premature ovarian insufficiency: within different clinical stages and different etiologies. Front Endocrinol. 2021;12:601752. 10.3389/fendo.2021.6017524. 10.3389/fendo.2021.601752 PMC8015703 33815272

[4] Vyas M, Simbo D, Mursalin M, Mishra V, Bashary R, Khatik G. Drug delivery approaches for doxorubicin in the management of cancers. Curr Cancer Therapy Reviews. 2019;16. 10.2174/1573394716666191216114950.

[5] Spears N, Lopes F, Stefansdottir A, et al. Ovarian damage from chemotherapy and current approaches to its protection. Hum Reprod Update. 2019;25(6):673–93. 10.1093/humupd/dmz027. 31600388 10.1093/humupd/dmz027 PMC6847836

[6] Buzun K, Bielawska A, Bielawski K, Gornowicz A. DNA topoisomerases as molecular targets for anticancer drugs. J Enzyme Inhib Med Chem. 2020;35(1):1781–99. 10.1080/14756366.2020.1821676. 32975138 10.1080/14756366.2020.1821676 PMC7534307

[7] Tacar O, Sriamornsak P, Dass CR. Doxorubicin: an update on anticancer molecular action, toxicity and novel drug delivery systems. J Pharm Pharmacol. 2013;65(2):157–70. 10.1111/j.2042-7158.2012.01567.x. 23278683 10.1111/j.2042-7158.2012.01567.x

[8] Zhang S, Zhu D, Mei X, Li Z, Li J, Xie M, et al. Advances in biomaterials and regenerative medicine for primary ovarian insufficiency therapy. Bioact Mater. 2021;6:1957–72. 10.1016/j.bioactmat.2020.12.008. 33426370 10.1016/j.bioactmat.2020.12.008 PMC7773538

[9] Marjoribanks J, Farquhar C, Roberts H, Lethaby A, Lee J. Long-term hormone therapy for perimenopausal and postmenopausal women. Cochrane Database Syst Rev. 2017;1(1):CD004143. 10.1002/14651858.CD004143.pub5. 28093732 10.1002/14651858.CD004143.pub5 PMC6465148

[10] Liang Y, Jiao H, Qu L, Liu H. Association between hormone replacement therapy and development of endometrial cancer: results from a prospective US cohort study. Front Med Lausanne. 2022;8:802959. 10.3389/fmed.2021.802959. 35111783 10.3389/fmed.2021.802959 PMC8801732

[11] Poluzzi E, Piccinni C, Raschi E, Rampa A, Recanatini M, De Ponti F. Phytoestrogens in postmenopause: the state of the Art from a chemical, Pharmacological and regulatory perspective. Curr Med Chem. 2014;21(4):417–36. 10.2174/09298673113206660297. 24164197 10.2174/09298673113206660297 PMC3963458

[12] Farkas S, Szabó A, Hegyi AE, Török B, Fazekas CL, Ernszt D, et al. Estradiol and estrogen-like alternative therapies in use: the importance of the selective and non-classical actions. Biomedicines. 2022;10:861. 10.3390/biomedicines10040861. 35453610 10.3390/biomedicines10040861 PMC9029610

[13] Desmawati D, Sulastri D. Phytoestrogens and their health effect. Open Access Maced J Med Sci. 2019;7(3):495–9. 10.3889/oamjms.2019.044. 30834024 10.3889/oamjms.2019.086 PMC6390141

[14] Tang Z, Zhang Q. The potential toxic side effects of flavonoids. BIOCELL. 2022;46(2):357–66. 10.32604/biocell.2022.015958.

[15] Wyse J, Latif S, Gurusinghe S, McCormick J, Weston LA, Stephen CP. Phytoestrogens. A review of their impacts on reproductive physiology and other effects upon grazing livestock. Animals. 2022;12(19):2709. 10.3390/ani12192709. 36230450 10.3390/ani12192709 PMC9559698

[16] Salehi B, Mishra AP, Nigam M, et al. Resveratrol: a double-edged sword in health benefits. Biomedicines. 2018;6(3):91. 10.3390/biomedicines6030091. 30205595 10.3390/biomedicines6030091 PMC6164842

[17] Zhang Y, Liu X, Zheng Z, et al. Network pharmacology uncovers that secoisolariciresinol diglucoside ameliorate premature ovarian insufficiency via PI3K/Akt pathway. Sci Rep. 2025;15(1):1493. 10.1038/s41598-024-83484-3. 39788972 10.1038/s41598-024-83484-3 PMC11717958

[18] Elsayed SH, Fares NH, Elsharkawy SH, Mahmoud YI. Flaxseed lignans alleviates isoproterenol-induced cardiac hypertrophy by regulating myocardial remodeling and oxidative stress. Ultrastruct Pathol. 2023;1–8. 10.1080/01913123.2023.2175944. 10.1080/01913123.2023.2175944 36789548

[19] Polat Kose L, Gulcin İ. Evaluation of the antioxidant and antiradical properties of some phyto and mammalian lignans. Molecules. 2021;26(23):7099. 10.3390/molecules26237099. 34885681 10.3390/molecules26237099 PMC8659077

[20] Osmakov DI, Kalinovskii AP, Belozerova OA, Andreev YA, Kozlov SA. Lignans as pharmacological agents in disorders related to oxidative stress and inflammation: chemical synthesis approaches and biological activities. Int J Mol Sci. 2022;23(11):6031. 10.3390/ijms23116031. 35682715 10.3390/ijms23116031 PMC9181380

[21] Jang WY, Kim MY, Cho JY. Antioxidant, anti-inflammatory, anti-menopausal, and anti-cancer effects of lignans and their metabolites. Int J Mol Sci. 2022;23:15482. 10.3390/ijms232415482. 36555124 10.3390/ijms232415482 PMC9778916

[22] Bedell S, Nachtigall M, Naftolin F. The pros and cons of plant estrogens for menopause. J Steroid Biochem Mol Biol. 2014;139:225–36. 10.1016/j.jsbmb.2012.12.004. 23270754 10.1016/j.jsbmb.2012.12.004

[23] Asselin CY, Lam A, Cheung DYC, et al. The cardioprotective role of flaxseed in the prevention of Doxorubicin- and Trastuzumab-mediated cardiotoxicity in C57BL/6 mice. J Nutr. 2020;150(9):2353–63. 10.1093/jn/nxaa144. 32510147 10.1093/jn/nxaa144

[24] Morsy MA, El-Sheikh AAK, Ibrahim ARN, Venugopala KN, Kandeel M. In silico and in vitro identification of secoisolariciresinol as a re-sensitizer of P-glycoprotein-dependent doxorubicin-resistance NCI/ADR-RES cancer cells. PeerJ. 2020;8:e9163. 10.7717/peerj.9163. 32566390 10.7717/peerj.9163 PMC7293189

[25] Byers SL, Wiles MV, Dunn SL, Taft RA. Mouse estrous cycle identification tool and images. PLoS ONE. 2012;7:e35538. 10.1371/journal.pone.0035538. 22514749 10.1371/journal.pone.0035538 PMC3325956

[26] Kropp J, Roti Roti EC, Ringelstetter A, Khatib H, Abbott DH, Salih SM. Dexrazoxane diminishes doxorubicin-induced acute ovarian damage and preserves ovarian function and fecundity in mice. PLoS ONE. 2015;10(11):e0142588. 10.1371/journal.pone.0142588. 26544188 10.1371/journal.pone.0142588 PMC4636352

[27] Adolphe JL, Whiting SJ, Juurlink BH, Thorpe LU, Alcorn J. Health effects with consumption of the flax lignan secoisolariciresinol diglucoside. Br J Nutr. 2010;103(7):929–38. 10.1017/S0007114509992753. 20003621 10.1017/S0007114509992753

[28] Purdue University. Mouse breeding colony management (Standard of Care SC-31-103). Purdue University, Institutional Animal Care and Use Committee (PACUC). 2019. https://www.purdue.edu/research/oevprp/regulatory-affairs/animal-research/docs/PU-SC-31-103%20Mouse%20Breeding%20Colonies.pdf

[29] Park HS, Chugh RM, Elsharoud A, Ulin M, Esfandyari S, Aboalsoud A, et al. Safety of intraovarian injection of human mesenchymal stem cells in a premature ovarian insufficiency mouse model. Cell Transplant. 2021;30:963689720988502. 10.1177/0963689720988502. 33593078 10.1177/0963689720988502 PMC7894598

[30] Biondo LA, Lima Junior EA, Souza CO, Cruz MM, Cunha RDC, Alonso-Vale MI, et al. Impact of doxorubicin treatment on the physiological functions of white adipose tissue. PLoS ONE. 2016;11:e0151548. 10.1371/journal.pone.0151548. 27015538 10.1371/journal.pone.0151548 PMC4807778

[31] Favreau-Lessard AJ, Blaszyk H, Jones MA, Sawyer DB, Pinz IM. Systemic and cardiac susceptibility of immune compromised mice to doxorubicin. Cardio-Oncol. 2019;5:2. 10.1186/s40959-019-0037-6. 10.1186/s40959-019-0037-6 PMC7048100 32154009

[32] Niringiyumukiza JD, Cai H, Chen L, Li Y, Wang L, Zhang M, et al. Protective properties of glycogen synthase kinase-3 inhibition against doxorubicin-induced oxidative damage to mouse ovarian reserve. Biomed Pharmacother. 2019;116:108963. 10.1016/j.biopha.2019.108963. 31125824 10.1016/j.biopha.2019.108963

[33] Herrero Y, Velázquez C, Pascuali N, May M, Abramovich D, Scotti L, et al. Resveratrol alleviates doxorubicin-induced damage in mice ovary. Chem Biol Interact. 2023;376:110431. 10.1016/j.cbi.2023.110431. 36925030 10.1016/j.cbi.2023.110431

[34] Zhang S, Liu Q, Chang M, Pan Y, Yahaya BH, Liu Y, et al. Chemotherapy impairs ovarian function through excessive ROS-induced ferroptosis. Cell Death Dis. 2023;14(5):340. 10.1038/s41419-023-05859-0. 37225709 10.1038/s41419-023-05859-0 PMC10209065

[35] Roti Roti EC, Leisman SK, Abbott DH, Salih SM, et al. Acute doxorubicin insult in the mouse ovary is cell- and follicle-type dependent. PLoS ONE. 2012;7(8):e42293. 10.1371/journal.pone.0042293. 22876313 10.1371/journal.pone.0042293 PMC3410926

[36] Gao Y, Wu T, Tang X, et al. Increased cellular senescence in doxorubicin-induced murine ovarian injury: effect of senolytics. Geroscience. 2023;45(3):1775–90. 10.1007/s11357-023-00728-2. 36648735 10.1007/s11357-023-00728-2 PMC10400526

[37] Han J, Wang H, Zhang T, Chen Z, Zhao T, Lin L, et al. Resveratrol attenuates doxorubicin-induced meiotic failure through inhibiting oxidative stress and apoptosis in mouse oocytes. Aging. 2020;12(9):7717–28. 10.18632/aging.103061. 32352929 10.18632/aging.103061 PMC7244048

[38] Hall JE. Endocrinology of the menopause. Endocrinol Metab Clin North Am. 2015;44(3):485–96. 10.1016/j.ecl.2015.05.010. 26316238 10.1016/j.ecl.2015.05.010 PMC6983294

[39] Parker R, Mathis C. Reproductive tract anatomy and physiology of the cow. Guide B-212. Reviewed by Turner J. College of Agricultural, Consumer and Environmental Sciences, New Mexico University. 2014. Accessed November 21, 2024.

[40] Nishi K, Gunasekaran VP, Arunachalam J, Ganeshan M. Doxorubicin-induced female reproductive toxicity: an assessment of ovarian follicular apoptosis, cyclicity and reproductive tissue histology in Wistar rats. Drug Chem Toxicol. 2018;41(1):72–81. 10.1080/01480545.2017.1307851. 28441888 10.1080/01480545.2017.1307851

[41] Ramya S, Poornima P, Jananisri A, Geofferina IP, Bavyataa V, Divya M, et al. Role of hormones and the potential impact of multiple stresses on infertility. Stresses. 2023;3(2):454–74. 10.3390/stresses3020033.

[42] Roti Roti EC, Ringelstetter AK, Kropp J, Abbott DH, Salih SM. Bortezomib prevents acute doxorubicin ovarian insult and follicle demise, improving the fertility window and pup birth weight in mice. PLoS ONE. 2014;9(9):e108174. 10.1371/journal.pone.0108174. 25251158 10.1371/journal.pone.0108174 PMC4176970

[43] European Society for Human Reproduction and Embryology (ESHRE) Guideline Group on POI, Webber L, Davies M, Anderson R, Bartlett J, Braat D, et al. ESHRE guideline: management of women with premature ovarian insufficiency. Hum Reprod (Oxford England). 2016;31(5):926–37. 10.1093/humrep/dew027. 10.1093/humrep/dew027 27008889

[44] Pourjafari F, Haghpanah T, Palmerini MG, Ezzatabadipour M. Serum scavenging capacity and folliculogenesis impact following flaxseed consumption in the first-generation mice pups. J Toxicol. 2022;5342131. 10.1155/2022/5342131. 35677062 10.1155/2022/5342131 PMC9170434

[45] Parikh M, Maddaford TG, Austria JA, Aliani M, Netticadan T, Pierce GN. Dietary flaxseed as a strategy for improving human health. Nutrients. 2019;11(5):1171. 10.3390/nu11051171. 31130604 10.3390/nu11051171 PMC6567199

[46] Jelodar G, Masoomi S, Rahmanifar F. Hydroalcoholic extract of flaxseed improves polycystic ovary syndrome in a rat model. Iran J Basic Med Sci. 2018;21(6):645–50. 10.22038/IJBMS.2018.25778.6349. 29942457 10.22038/IJBMS.2018.25778.6349 PMC6015245

[47] Abd El-Galil MM, Mohammed FS. The possible effect of flaxseed extract on letrozole-induced polycystic ovary rat model: correlative histological and functional study. Al Azhar Med J. 2021;50:3055–100. 10.21608/amj.2021.196448.

[48] He X, Wang Y, Wu M, Wei J, Sun X, Wang A, et al. Secoisolariciresinol diglucoside improves ovarian reserve in aging mouse by inhibiting oxidative stress. Front Mol Biosci. 2021;8:806412. 10.3389/fmolb.2021.806412. 35059437 10.3389/fmolb.2021.806412 PMC8764264

[49] Paul A, Sujatha K, Srilatha CH, Kumar V. Amelioration of lead induced toxicity on rat ovary with *Linum usitatissimum* (flaxseed) and *Emblica officinalis* (Amla). PIJ. 2021;10(8):1124–30.

[50] Chen Y, Yang Z, Bai J, Wang X, Yuan Q, Mi Y, et al. Bioactive lignan Honokiol alleviates ovarian oxidative stress in aging laying chickens by regulating SIRT3/AMPK pathway. Antioxidants. 2024. 10.3390/antiox13030377. 38539910 10.3390/antiox13030377 PMC10967992

[51] Yaşar P, Ayaz G, User SD, Güpür G, Muyan M. Molecular mechanism of estrogen-estrogen receptor signaling. Reprod Med Biol. 2017;16(1):4–20. 10.1002/rmb2.12006. 29259445 10.1002/rmb2.12006 PMC5715874

[52] Tang Z-R, Zhang R, Lian Z-X, Deng S-L, Yu K. Estrogen-receptor expression and function in female reproductive disease. Cells. 2019;8(10):1123. 10.3390/cells8101123. 31546660 10.3390/cells8101123 PMC6830311

[53] Sharma C, Vani V, Jayamma Y, Inamdar LS. Estrous cycle in rodents: phases, characteristics, and neuroendocrine regulation. J Sci. 2020;51:40–53.

[54] Tanideh R, Delavari S, Farshad O, Irajie C, Yavari Barhaghtalab MJ, Koohpeyma F, et al. Effect of flaxseed oil on biochemical parameters, hormonal indexes, and Stereological changes in ovariectomized rats. Vet Med Sci. 2021;7(2):521–33. 10.1002/vms3.372. 33103380 10.1002/vms3.372 PMC8025639

[55] Abouelmagd A, Hassan G. Hormonal and metabolic benefits of flaxseed oil in ovariectomized rats. Bull Egypt Soc Physiol Sci. 2023;43(1):33–46. 10.21608/besps.2022.152592.1127.

[56] Dutra PA, Pinto LFB, Cardoso Neto BM, Gobikrushanth M, Barbosa AM, Barbosa LP, et al. Flaxseed improves embryo production in Boer goats. Theriogenology. 2019;127:26–31. 10.1016/j.theriogenology.2018.12.038. 30639693 10.1016/j.theriogenology.2018.12.038

[57] Didarkhah M, Vatandoost M, Dirandeh E, Dadashpour Davachi N. Effects of flaxseed-rich diet on reproductive performance in estrous-synchronized Baluchi ewes. Arch Razi Inst. 2020;75(3):397–404. 10.22092/ari.2020.341899.1442. 33025780 10.22092/ari.2020.341899.1442 PMC8418811

[58] Gotardo AT, Dipe VV, Hueza IM, Górniak SL. Maternal feed restriction during pregnancy in Wistar rats: evaluation of offspring using classical and immunoteratology protocols. Hum Exp Toxicol. 2017;36(6):603–15. 10.1177/0960327116660750. 27496853 10.1177/0960327116660750

[59] Ortega MA, Fraile-Martínez O, García-Montero C, Sáez MA, Álvarez-Mon MA, Torres-Carranza D, et al. The pivotal role of the placenta in normal and pathological pregnancies: a focus on preeclampsia, fetal growth restriction, and maternal chronic venous disease. Cells. 2022;11(3):568. 10.3390/cells11030568. 35159377 10.3390/cells11030568 PMC8833914

[60] Abulé RM, Bernardes LS, Doro GF, Miyadahira S, Francisco RP. Reduced placental volume and flow in severe growth restricted fetuses. Clinics. 2016;71(6):332–7. 10.6061/clinics/2016(06)08. 27438567 10.6061/clinics/2016(06)08 PMC4930658

